# Tocilizumab for the fifth progression of cystic childhood craniopharyngioma—a case report

**DOI:** 10.3389/fendo.2023.1225734

**Published:** 2023-10-11

**Authors:** Evelien de Vos-Kerkhof, Dennis R. Buis, Maarten H. Lequin, Carlien A. Bennebroek, Eleonora Aronica, Esther Hulleman, Nitash Zwaveling-Soonawala, Hanneke M. van Santen, Antoinette Y. N. Schouten-van Meeteren

**Affiliations:** ^1^ Department of Neuro-oncology, Princess Máxima Center, Utrecht, Netherlands; ^2^ Department of Neurosurgery, University of Amsterdam, Amsterdam University Medical Center (UMC), Amsterdam, Netherlands; ^3^ Department of Radiology, University Medical Center Utrecht, Utrecht, Netherlands; ^4^ Department of Ophthalmology, Amsterdam University Medical Center (UMC), Amsterdam, Netherlands; ^5^ Department of Neuropathology, University of Amsterdam, Amsterdam University Medical Center (UMC), Amsterdam, Netherlands; ^6^ Department of Pediatric Endocrinology, Amsterdam University Medical Center (UMC), Amsterdam, Netherlands; ^7^ Department of Pediatric Endocrinology, Wilhelmina Children’s Hospital, University Medical Center Utrecht, Utrecht, Netherlands

**Keywords:** adamantinomatous craniopharyngioma, cyst, tocilizumab, visual impairment, hypothalamus, case report

## Abstract

We present the case of a 15-year-old girl, with a fifth cystic progression of an adamantinomatous craniopharyngioma after multiple surgeries and previous local radiotherapy. She had severe visual impairment, panhypopituitarism including diabetes insipidus, and several components of hypothalamic damage, including morbid obesity and severe fatigue. To prevent further late effects hampering her quality of survival, she was treated biweekly with intravenous tocilizumab, an anti-interleukin-6 agent, which stabilized the cyst for a prolonged time. Based on the biology of adamantinomatous craniopharyngioma, this immune-modulating treatment seems promising for the treatment of this cystic tumor in order to reduce surgery and delay or omit radiotherapy.

## Introduction

Craniopharyngioma is a rare WHO grade I brain tumor in the supratentorial midline deriving from the epithelial cells remaining in embryonal development from Rathke’s pouch (1). The subtype adamantinomatous craniopharyngioma (ACP) contains solid and cystic components and is characterized by mutations in CTNNB1 (encoding β-catenin) and typically occurs in children (2). Despite good survival, craniopharyngioma is located in a crucial part of the brain. Consequently, patients may struggle with visual impairment, endocrine deficiencies, hypothalamic damage, morbid obesity, severe lack of energy, and neurocognitive and/or psychosocial consequences (3, 4). Repeated neurosurgery and radiotherapy are limited due the risk of damage of many vulnerable structures in the suprasellar region of the tumor (5, 6).

We present the case of a 15-year-old girl with multiple cystic progressions of ACP limiting local therapeutic possibilities and describe the results of systemic tocilizumab therapy.

## Case report

**Figure 1:**
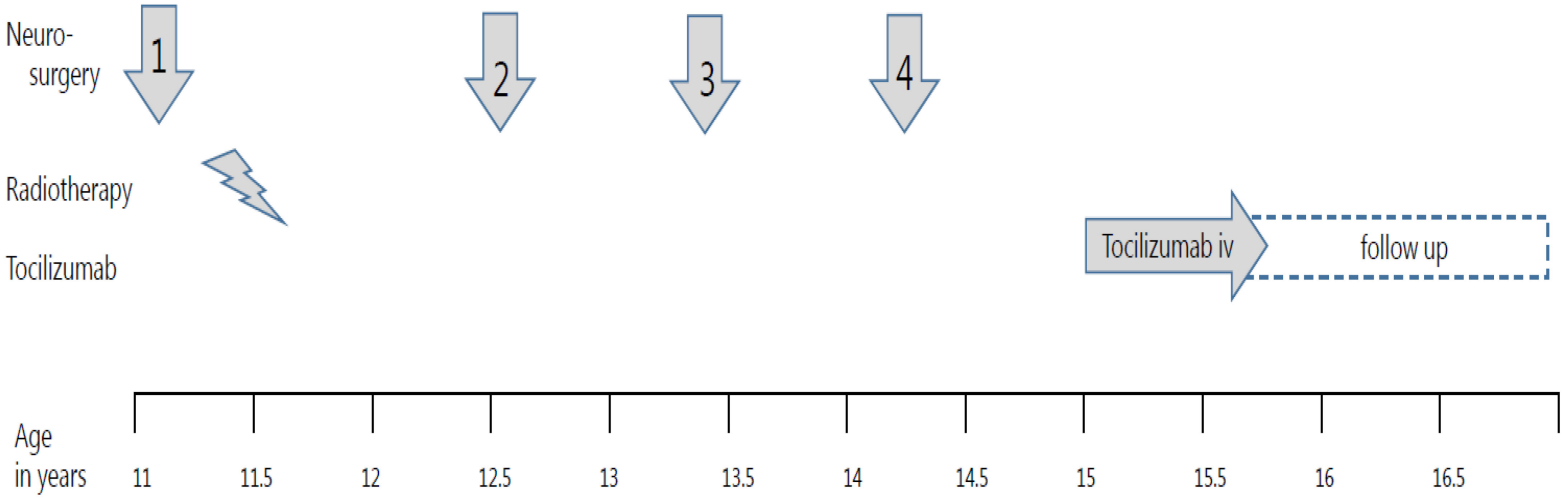
Treatment timeline in a girl with progressive adamantinomatous craniopharyngioma. The timeline for treatment interventions for progressieve adamaninomatous craniopharyngioma from 11 till 16,5 years old is shown in the graph. Four neurosurgical interventions are shown with arrows, while the radiotherapy period is depicted with a lightning bold pictogram. The systemic treatment was composed of biweekly 800 mg tocilizumab intravenously.

A 15-year-old girl showed ongoing cystic progression of residual ACP with treatment interventions shown in **Figure 1**. Her initial presentation was at 11 years of age, in the emergency room of a general hospital with severe complaints of headache and vomiting. The family history was negative for brain tumors. Previously, she was diagnosed with growth retardation, with a decrease in height standard deviation (SD) from 0 SD to −2SD. Her psychomotor development was normal with excellent school results, without visual impairment or history of ocular abnormalities. A CT scan of the brain demonstrated a large suprasellar mass growing into the third ventricle causing biventricular enlargement. She developed convulsions and became obtunded, after which she was intubated. She was transferred to the neurosurgery department of a university medical center for release of the hydrocephalus by external ventricular drainage.

**Figure 2:**
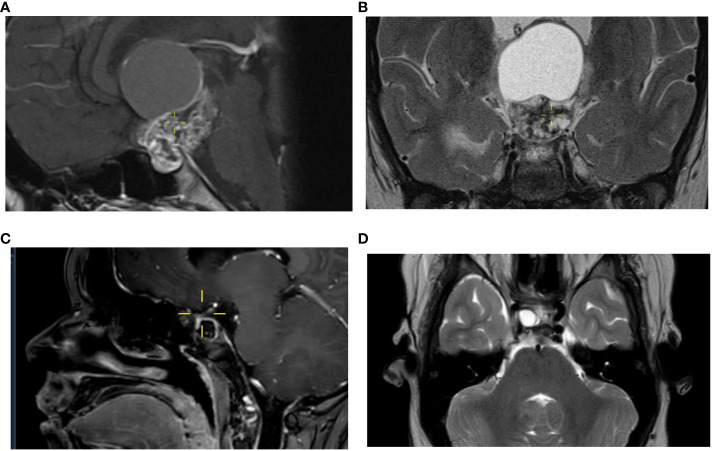
Course of craniopharyngioma on MR imaging. The MRI at diagnosis performed in a child of 11 years old shows a suprasellar and sellar cystic-solid lesion with calcifications, measuring a maximum of 5,2 cm in cranio-caudal direction visible on a sagittal image, a T1 3 mm 0,3 gap with gadolinium **(A)** and on a coronal image, T2 TSE 3 mm 0,3 gap **(B)** consistent with the diagnosis of a craniopharyngeoma. After four previous neurosurgical resections a cystic lesion close to the right optic nerve increases up to 1,1 cm visible on a sagittal image after contrast **(C)** and on the axial image T2 TSE, 3 mm without gap **(D)**.

**Figure 3:**
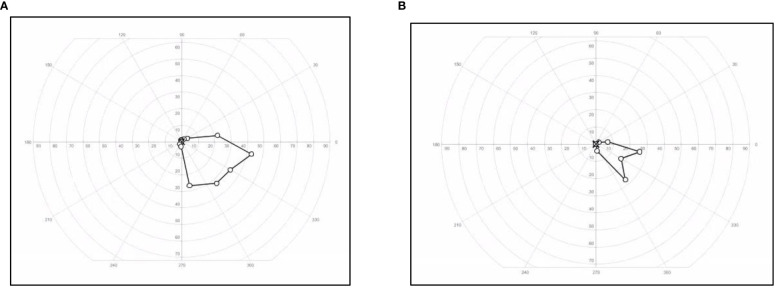
Visual field changes in a patient with cystic craniopharyngioma. Visual field of the left eye restricted to the inferionasal kwadrant measured with Goldman method at start of treatment **(A)** with tocilizumab and at end of treatment **(B)**.

MR imaging demonstrated a partially cystic and solid tumor suspected of craniopharyngioma, although chiasmatic glioma and intra-cranial germ cell tumor were in the differential diagnosis. The size was 5.2 cm craniocaudal × 3.3 cm anterior–posterior growing into the third ventricle, infiltrating the hypothalamus, compressing the thalamus, and stretching the chiasm anterior and downwards (**Figures 2A, B**). She was started on stress doses of hydrocortisone, anticipating pituitary hormone deficiencies due to pituitary stalk compression and hypothalamic involvement of the tumor. The tumor was neurosurgically partially resected, and histology revealed ACP. A 1-day post-operative MRI showed a small intrasellar remnant of 1.3 cm × 0.7 cm × 0.8 cm. The postoperative course was complicated by central diabetes insipidus and sodium shifts ranging from 115 mmol/L to 154 mmol/L. For central hypothyroidism, supplementation with thyroid hormone was started. She also suffered from a temporary right-sided paresis due to osmotic demyelination. Post-operatively, her vision deteriorated with an episode of complete blindness, where after the right eye remained without light perception and in the left eye, a best corrected visual acuity (BCVA) of 0.16 decimal (LogMAR, 0.8) was measured combined with a large visual field defect represented by an absolute temporal hemifield and superonasal deficit (**Figure 3A**).

The first cystic progression of the craniopharyngioma occurred after 4 months when the MRI showed cysts up to 1.6 cm in diameter. No clinical signs occurred; the Lansky score was 80. Growth hormone therapy had not been started yet. This cystic progression was the reason to perform local radiotherapy with 54 Gy at the age of 11.5 years. The cystic progression re-occurred up to 1.4 × 2.0 × 2.4 cm with a crucial location in the proximity of the chiasm, leading to compression of the chiasm.

To prevent further deterioration of her severely reduced vision, repeated neurosurgical interventions were deemed necessary. At 12.5 years of age, a transsphenoidal neurosurgical attempt at complete resection was made; however, a fibrotic remnant in the left cavernous sinus could not be removed. At 13.3 years of age, a transcranial third subtotal resection was performed for the recurrent cystic part of the tumor. Large amounts of cystic parts of the tumor were removed; however, suspected intrasellar tumor remnants could still not be reached. At 13.7 years of age, a ventriculoperitoneal shunt was placed for complaints of reduced vision due to communicating hydrocephalus without tumor growth. After placement of the VP shunt, the vision improved to her baseline after first surgery. At 14 years of age, a fourth subtotal resection was performed via a combined left transcranial and transsphenoidal approach. The tumor was found to be severely adherent to all vascular and nerve structures of the skull base. One tumor remnant under the functional left optic nerve was not deemed resectable without harming this optic nerve and possibly causing further visual deterioration; therefore, this remnant was left in place.

**Figure 4:**
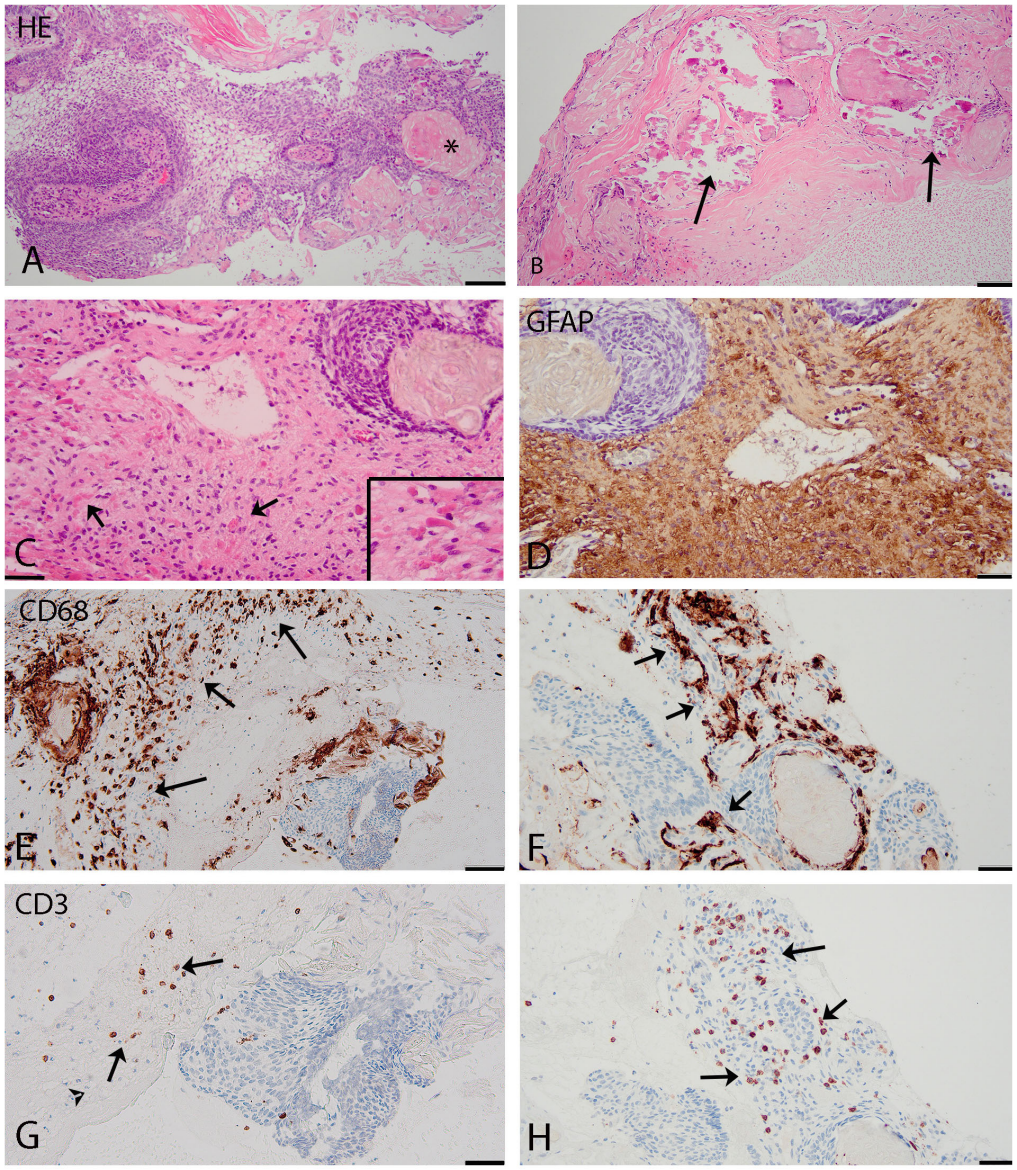
**(A–C)** (HE): histological features adamantinomatous craniopharyngioma. **(A)**: squamous epithelial tumour with basal palisading of tumour cells, stellate reticulum, and wet keratin (asterisk). **(B)**: prominent calcifications in the cyst wall (arrows). **(C)**: interface of adamantinomatous craniopharyngioma and brain tissue with Rosenthal fibers present in the reactive piloid gliosis surrounding the tumor (arrows and insert). **(D–H)**: Immunohistochemistry. **(D)** (GFAP) shows reactive piloid gliosis. **(E, F)** (CD68) shows macrophages (arrows) in the cyst wall **(E)** and around the tumor **(F)**. **(G, H)** (CD3): T-lymphocytes (arrows) located around and focally **(H)** within the tumor. Scale bars: **(A–C)**, 100 µm; **(D–H)**, 50 µm.

Extensive histopathological analysis of the ACP was performed with the tissue of the latest surgery showing confluent epithelial fields of multilayer and matured squamous epithelial cells with basal palisades and variable degrees of keratinization and focal calcification (**Figure 4**). The epithelium showed extensive spongiosis with the formation of microcystic regions (stellate reticulum). Whole exome sequencing analysis and RNA sequencing showed a CTNBB1 mutation in exon 3 (c.98CT, p.S33F) in 34% of the reads and no relevant mutations in exons 11 and 15 of the *BRAF* gene. Overexpression of FGF3/FGF4 N was detected with a Z-score of 4.004/5.598 compared to the other samples in the database. There was a low mutational burden, and no mRNA expression of Programmed death-ligand 1 (PD-L1) was observed.

Over the years, hypothalamic damage became increasingly apparent, as evidenced by morbid obesity with BMI increasing up to 36.5 kg/m^2^ despite strict dietary regulations. At the age of 12 years, her resting energy expenditure was only 51% of the expected value in 2019. She had panhypopituitarism with diabetes insipidus but with adequate thirst regulation. She suffered from temperature dysregulation with extreme color changes of the hands, feet, and cheeks, and neuropathic pain required treatment with amitriptyline. Her sleeping pattern was disturbed with repeated nightly awakenings. Her energy level was very low, partially based on the hypothalamic damage in combination with acquired brain injury Lansky score, which was stable at 80. Her low vision was highly burdensome, and therefore, she switched to a school for visually impaired and blind children, where she could follow age-appropriate high school with good results.

Because of the slight decrease in the BCVA of her left eye to 0.1 decimal (1.0 LogMAR) combined with the subsequent fifth progression with new cystic growth until 0.9 cm × 1.0 cm × 1.0 cm (**Figures 2C, D**) at 15 years of age, we decided to start experimental therapy with the anti-interleukin-6 agent tocilizumab (TCZ) (20 mg/ml) 800 mg every 2 weeks intravenously during 1 h. This led to the stabilization of the cyst. The TCZ was tolerated well with no direct side effects, including stable blood counts, creatinine levels, liver function test, and triglyceride levels. No unanticipated events occurred. After a total treatment period of 9 months, the TCZ was ended. Despite the TCZ treatment, longitudinal analyses of visual functions showed a small decrease in BCVA to 1.2 LogMAR and an additional constriction of her visual field to an inferonasal remnant (**Figure 3B**). According to the WHO criteria of visual impairment, she is considered severely visually impaired (https://icd.who.int/browse10/2019/en#/H54.9) with dependence on visual aids.

The patient and parents experienced no significant negative side effects besides a stiff arm for 1–2 days. Additionally, they experienced that desmopressin for diabetes insipidus seemed less effective within the first 24 h after each treatment day. They experienced a further lack of energy during the 9-month treatment, which was, however, already present in her pre-existent medical status. Furthermore, it was a mentally challenging period, since it was uncertain whether the treatment would be effective and how long the treatment would be necessary. In the end, they are very pleased that the therapy appears to have had an inhibitory impact on the tumor cyst.

Before the initiation of TCZ, there were four episodes of cystic growth of ACP with maximum intervals of 9 months after each neurosurgical procedure. To date, from the start of TCZ, both the residual cystic and solid components of the craniopharyngioma have remained stable with no requirement for further local treatment for almost 3 years until now.

We suggest that treatment with TCZ has effectively stabilized cystic parts of ACP in our patient and prevent a fifth tumor surgery in this vulnerable and complex suprasellar area; however, there was a mild decrease in visual function. Future cohort studies on treatment of ACP with TCZ are needed to confirm our experience.

## Discussion

Our case report illustrates TCZ’s possible effectiveness in interrupting ACP’s repeated cystic growth. This phenomenon may offer new insight into the possibility of systemic treatment of cystic ACP.

Neurosurgical resection is the common approach for the treatment of ACP; however, the optic chiasm, pituitary, hypothalamus, and central arteries are delicate structures in the cerebral midline, hindering safe neurosurgical gross total resection (5). The role of neurosurgery is indispensable. Gross total resection may be curative, but due to the involvement of neurovascular structures, the optic pathways, and (hypo-)thalamic structures complete resection may come at a price. In a recent systematic review, the most common complications seen within a mean follow-up duration of 78 months after surgery for craniopharyngioma included visual impairment (27,2%), need for hormonal replacement therapy (63%), obesity (25%), diabetes insipidus (55%), cranial nerve palsy (14%), vascular injury (15%), and hydrocephalus, meningitis, and CSF-leak in 6%, 5%, and 7%, respectively (7). Besides the key role of neurosurgery, radiation therapy is considered an effective treatment strategy (6, 8, 9).

Although 85%–90% of patients have long-term survival, the chance of long-term morbidities after craniopharyngioma and local therapies is high (3). The major consequences of ACP are visual impairment, endocrine deficiencies, hypothalamic morbidity, severe fatigue, and acquired brain injury with neurocognitive and psychosocial changes (1, 10). In the longer term, cardiovascular risk can lead to cerebrovascular infarcts after radiation of the cerebral arteries and also myocardial infarction with severe obesity (11).

In the past decades, searching for an effective systemic therapy to treat craniopharyngioma has been ongoing. Interferon-alpha was applied as intra-cystic treatment striving for immunomodulatory effects. It is thought that this therapy plays a role in both generating an immunological response via immune cell activation and in the promotion of cellular differentiation and inhibition of proliferation by modulating signaling mechanisms (12–14). Intra-cystic interferon led to a clinical and radiological response in 76% of children and could delay further therapy for a median of 5.8 years in 56 out of 60 children (15, 16). In addition, subcutaneous interferon in a phase II trial improved PFS in children with cystic ACP lesions (17).

The main biological background of ACP involves the WNT-signaling pathway with mutations in CTNNB1 (encoding β-catenin) (2). Furthermore, heterogeneous components of ACP are found in single-cell RNA analysis with four meta-signatures related to epithelial development, Wnt signaling, cell cycle progression, and stress responses with cellular activation. The latest has been related to inflammation in the tumor microenvironment of ACP (**Figure 4**) (18). Focusing on inflammatory parameters in the extension of interferon therapy, we learned more about the proinflammatory environment and the complex paracrine signaling in ACP. ACP cells expressed PD-L1 predominantly in the cyst line while also revealing tumor cell–intrinsic PD-1 expression in whorled epithelial cells with nuclear-localized beta-catenin and showing elevated mammalian target of rapamycin (mTOR) and mitogen-activated protein kinase (MAPK) signaling and activated CD8+ T cells (19–22). Inhibition of this pathway in human and murine ACP tumor tissue *ex vivo* reduces proliferation and increases apoptosis. This may suggest that targeting this pathway can be therapeutic for ACP, while PD-L1 and/or PD-1 might be relevant immunotherapeutic targets.

ACP cells are secreting inflammatory mediators, including interleukin (IL)1A and IL-6. IL6 is seen in ACP cysts at a high level in comparison with the cysts of pilocytic astrocytoma. This suggests an active role of this interleukin in the cysts of ACP (23). Identifying these mediators, especially in cystic compartments, suggests a proinflammatory cascade and activation of the inflammasome (19, 24–27). The role of these inflammatory mediators on the tumor cell level is unknown. From cell culture experiments, it is thought that IL-6 promotes epithelial-to-mesenchymal transition and increases the migration of ACP cells (24). This transition is associated with ACP recurrence or progression and thus tumor growth (28). IL-6R is expressed both within tumor epithelia and reactive tissue (24, 27), and it was shown that also other inflammation markers as macrophages, microglia, and T lymphocytes are detectable in ACP (2, 29).

Tocilizumab is a humanized monoclonal antibody against soluble and membrane-bound IL-6R. It is indicated as a treatment for several autoimmune or inflammatory diseases, including systemic rheumatoid arthritis and juvenile idiopathic arthritis (30–33). It also treats cytokine release syndrome following chimeric antigen receptor (CAR) T-cell therapy for acute lymphoblastic leukemia (34). Two clinical trials with TCZ for ACP are ongoing: a single institution Phase 0/Feasibility Trial and a multicenter phase II trial (ACTEMRA NCT05233397) (35, 36).

In a previous report including two patients with cystic ACP, treatment with IL-6 receptor inhibition with TCZ showed a significant response with cystic regression. In those cases, therapy took 7 months in single TCZ treatment and 14 months in combination with bevacizumab (23). Our patient is now the third case with ACP described in the literature who was treated with TCZ. The profile of this ACP fitted two meta-signatures as described by Jiang et al., keratinized-like epithelium with stellate reticulum in the microcysts and whorl-like epithelium with Wnt features and activated mTOR pathway via over-expression of FGF3/FGF4 (18). The strength of this case is that earlier repeated cystic growth was evident and was interrupted successfully. The patient had benefitted from 9 months of TCZ treatment, which she tolerated very well, and for whom further local interventions have been deferred for almost 3 years since last neurosurgery. Lack of treatment data on more patients still remains a major limitation for regular clinical application of tocilizumab in aCP.

In conclusion, systemic therapy for ACP can be potentially helpful for tumor reduction or stabilization to refrain from further harmful local therapies in progressive disease. Future clinical research is necessary to define the duration of therapy and response. Clinical trials should also focus to identify which biological subtypes of ACP benefit from the diverse options for targeted systemic therapy.

## Data availability statement

The original contributions presented in the study are included in the article/supplementary material. Further inquiries can be directed to the corresponding author.

## Ethics statement

Written informed consent was obtained from the minor(s)’ legal guardian for the publication of any potentially identifiable images or data included in this article. The study was conducted in accordance with the local legislation and institutional requirements.

## Author contributions

All authors listed have made a substantial, direct, and intellectual contribution to the work and approved it for publication.
